# A Causal Layered Analysis of Oral Health Disparities and Policy Strategies for Vulnerable Iranian Populations

**DOI:** 10.34172/ijhpm.9245

**Published:** 2025-10-18

**Authors:** Shervan Shoaee, Afshin Sarafinejad

**Affiliations:** ^1^Elderly Health Research Center, Endocrinology and Metabolism Population Sciences Institute, Tehran University of Medical Sciences, Tehran, Iran.; ^2^Kerman Oral and Dental Diseases Research Center, Kerman University of Medical Sciences, Kerman, Iran.; ^3^Medical Informatics Research Center, Institute for Futures Studies in Health, Kerman University of Medical Sciences, Kerman, Iran.; ^4^Clinical Informatics Research and Development Lab, Clinical Research Development Unit, Shafa Hospital, Kerman University of Medical Sciences, Kerman, Iran.

## Introduction

 Oral health extends beyond healthy teeth, playing a critical role in overall well-being and quality of life. Poor oral health is strongly associated with general health, education, and productivity, highlighting the urgent need for global action to improve oral health as part of the Sustainable Development Goals. Particularly, it plays an essential role in achieving goals related to poverty alleviation, hunger reduction, quality education, gender equality, and overall well-being.^1-3^

 Addressing these challenges requires a policy shift, recognizing oral health as an integral component of national healthcare systems rather than an isolated clinical intervention.^3^

 Despite being largely preventable, dental caries and periodontal disease remain the most prevalent diseases globally, affecting billions of people and leading to substantial economic burdens and social inequalities.^4,5^ Although clear links exist between oral health and a range of systemic diseases like cardiovascular conditions, diabetes, respiratory infections, chronic kidney disease, and even increased mortality,^2,6^ oral health continues to receive inadequate attention in public health policy.

 Studies show that oral health literacy remains below satisfactory levels in over 60% of the global population, including Iran, limiting individuals’ ability to adopt preventive behaviors and seek timely care, disproportionately affecting vulnerable groups.^7,8^

 In Iran, more than 50% of those aged 65-74 years are completely edentulous according to national oral health surveys conducted during 2012-2013.^9^ The DMFT index (Decayed, Missing and Filled Teeth) among aged people was alarmingly high around 26.84; and approximately 92.5% of DMFT index was only related to missing teeth.^1,6,10^

 Individuals with disabilities face additional challenges, such as limited access to preventive oral care and specialized care providers, as well as communication and comorbidities issues.^3,11^ While Iran does not face a shortage of dentists, there is a significant lack of oral healthcare providers trained to meet the specific needs of the elderly and disabled populations.^10^

 The Golkhand Project successfully integrated preventive oral healthcare into Iran’s elderly and disabled care services under the Welfare Organization’s supervision, incorporating oral health guidelines into standard care protocols. After years of planning, oral health services were officially included in rehabilitation care centers since 2023 (Persian News),^12^ establishing oral care as a fundamental component of these services.

 Interprofessional and closer medical and dental and non-dental personnel collaboration, and in particular closed-loop referral process, have been experienced as reasons for successful integration.^13^ The FDI World Dental Federation highlights primary oral healthcare as a vital part of public health.^14^ To improve access to essential services, national strategies should combine government support and collaboration among organizations.^3^ The FDI White Paper stresses the need to integrate oral health into general healthcare systems and maintain strong policy efforts to ensure everyone can access necessary care.^14^

 The oral health status of a country’s elderly population, often serves as a reflection of the overall health status of its population, underscoring the urgent need for comprehensive strategies, like insurance coverage, targeting this group.^15^

 This paper employs causal layered analysis (CLA) as a framework for examining oral health disparities. CLA is a futures-oriented approach that explores issues across four interconnected levels: litany (surface concerns), systemic causes (structural factors), worldviews (underlying values), and myths/metaphors (deep cultural narratives) (Figure).^16^

**Figure:**
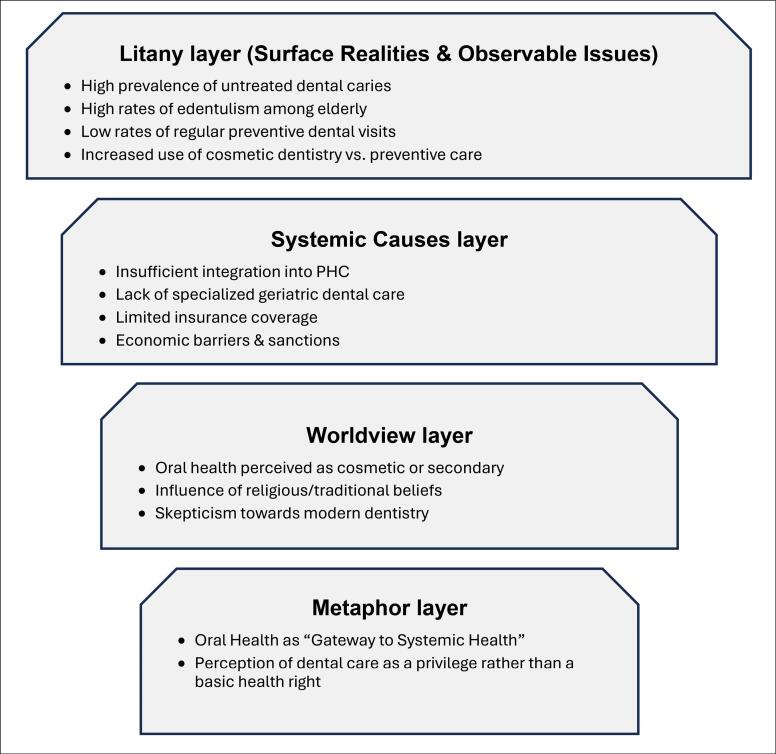
Multilayered Framework of Structural and Cultural Drivers of Oral Health Inequities in Iran. Abbreviation: PHC, primary healthcare.

 Unlike conventional approaches that focus mainly on observable problems, CLA reveals how cultural and structural narratives shape health behaviors and policies, making it particularly relevant for analyzing oral health disparities.

## Oral Health: A Multilayered Challenge Using Causal Layered Analysis Framework

###  A. Litany: Surface Realities

 Oral health literacy remains alarmingly low among vulnerable populations in Iran. Basic oral hygiene practices, such as using toothbrushes, dental floss, and regular dental check-ups, are uncommon, particularly in low-resource settings. In line with national assessments and the FDI World Dental Federation reports, this lack of awareness is deeply intertwined with gaps in national oral health strategies and their implementation at primary healthcare (PHC) levels. In Iran, oral hygiene practices remain insufficient, especially among the elderly and individuals with disabilities, leading to untreated conditions that exacerbate systemic health risks.^1,6^

###  B. Systemic Causes (Structural Level)

 Oral health disparities in Iran stem from deep rooted systemic barriers that hinder access, affordability, and prioritization within the healthcare system. These structural challenges demand multi-level interventions to address the gaps:

- Healthcare policy gaps: Despite nominal integration, oral health remains poorly implemented in PHC, particularly for vulnerable groups, with preventive services excluded from public insurance coverage.^13,15^
- Economic barriers: Economic sanctions, inflation, and currency devaluation have eroded household purchasing power and disrupted dental supply and materials and equipment chains, exacerbating out-of-pocket costs for care.^9^
- Education and prevention: Oral health education is neglected in schools and communities, while dental curricula lack focus on caring for elderly or disabled. Worse, preventive measures like fluoride therapy do not include these populations.^10,17^
- Urban-rural divide: Rural areas face significant disparities in access to dental care. Dentists and facilities cluster in urban centers, leaving rural regions underserved.^15^
- Cultural attitudes and traditional influences: Predominant perceptions treat oral health as cosmetic or even focus on extraction as a final decision. It shapes personal behaviour compounded by traditional remedies and religious practices (eg, fasting) that delay clinical care, most among older or more conservative populations.^5,18,19^
- Ineffective workforce expansion: The surge in dental schools and dentists during 2002 to 2014 and similar trend afterward, failed to improve the DMFT index, revealing that workforce growth alone is ineffective without preventive strategies.^1,9^

These systemic issues necessitate urgent policy reforms, equitable resource distribution, and preventive-focused integration into national healthcare.

###  C. Attitudes Toward Oral Health and its Role in Systemic Health 

 Societal perceptions of oral health as a secondary or cosmetic issue contribute significantly to its neglect in public health policy.^5,19^ This is particularly detrimental for vulnerable groups such as the elderly and individuals with disabilities, since the critical systemic impacts and quality-of-life effects of oral health are often underestimated by their caregivers, families, and policy-makers. This lack of prioritization is evident in national health agendas, where preventive care and tailored education programs remain limited.^14,15^

 Among vulnerable groups, low health literacy and limited education access lead to avoidable outcomes like tooth loss and periodontal disease, which impair nutrition, communication, and self-esteem while increasing risks of other diseases. Growing evidence confirms biological links between oral inflammation and systemic conditions, underscoring the need for integrated health strategies.^2,19^

 A paradigm shift in public perception and policy design is essential. National oral health programs must go beyond treatment and actively include preventive strategies and caregiver education tailored to the unique needs of elderly and disabled individuals.^15^

###  D. Metaphor: Reframing the Narrative 

 Oral health must be reimagined as the “gateway to systemic health.”^20^ Similarly, digital health tools, including teledentistry and AI-driven diagnostics, can serve as a “bridge” to connect underserved populations with essential oral health services.^21,22^ However, to truly integrate these tools into national health strategies, they must be embedded within a structured policy framework that ensures equitable access and governmental support. These tools not only improve accessibility but also ensure equity in oral healthcare, making it a practical and actionable goal.^23^

 Given these systemic and structural challenges, targeted policy interventions must address both immediate access issues and long-term sustainability of oral healthcare.^15^

## Policy Recommendations: A Path to Equitable Oral Health

###  Integrating Oral Health into Primary Healthcare 

 To address the issue of low integration of oral health into PHC, embedding preventive oral care into existing PHC systems can ensure universal access to essential services while addressing the specific needs of vulnerable populations.

 Collaboration between key stakeholders, including the Ministry of Health and Medical Education, the Welfare Organization, the Ministry of Education, and non-governmental organizations, is critical for achieving an integrated approach. Such collaboration can facilitate comprehensive care for the elderly and individuals with disabilities, ensuring accessibility and consistency.

 Establishing a multidisciplinary team for comprehensive geriatric assessment is essential. These teams should be established within PHC centers and include physicians, dentists, nurses, social workers, hygienists, psychologists, physiotherapists, and nutritionists, with oral health integrated into the routine comprehensive geriatric assessment process. Practical steps include a brief oral health screen, referral pathways to dental services, and basic caregiver education to support daily oral care.^13-15^ Furthermore, policy frameworks should incorporate oral health screening into routine geriatric and disabled assessments, ensuring early detection and intervention for oral diseases. Additionally, incorporating oral health into the ongoing education of specialists, general practitioners, and allied health professionals (such as physiotherapists and nutritionists) will help address the multifaceted needs of elderly and disabled populations. In parallel, specialized training and tailored curricula for dental and medical students can further strengthen the healthcare workforce’s capacity to meet these challenges.^10,20,23^

###  Targeted Oral Health Programs for Elderly and Disabled Populations 

 To address low oral health literacy and the lack of preventive behaviors among elderly populations, programs tailored to the unique needs of the elderly and individuals with disabilities should emphasize both direct services and empowerment through education. This includes rural and urban oral healthcare centers, portable dental care units (bus, van truck, etc), home-based dental care services, training caregivers, and developing specialized curricula for dental professionals to treat these populations effectively. It is noticeable that some of the community-based efforts were not that successful to improve oral health and also personal practical skills in daily oral hygiene for elderly people.^24^ Learning more and continuous education programs for caregivers can help in the formulation of better localized interventions.

###  Adding Digital Health Solutions 

 To mitigate the challenges of geographical accessibility and shortage of trained geriatric dentists, as mentioned before, using digital tools like teledentistry, remote consultations, AI-driven diagnostics, and mobile health apps can significantly improve service delivery for underserved elderly and disabled populations.^25,26^ In rural areas, however, the feasibility of these solutions depends on reliable internet connectivity, which has expanded in Iran in recent years but remains uneven across provinces. Moreover, smartphone ownership among the elderly population is relatively low compared with younger groups. To overcome these barriers, strategies may include subsidized devices, shared access through community health houses, and the development of simplified user interfaces. From a cost-effectiveness perspective, digital interventions are more sustainable when integrated into existing PHC and telemedicine infrastructures rather than being implemented as stand-alone programs.^14,25,26^ Implementation can be facilitated through teledentistry hubs within PHC facilities, integration with existing telemedicine platforms, and caregiver training to improve usability.

 There are evidences that emphasize the role of digital health in bridging gaps in service delivery.^25,26^ By integrating digital solutions into national oral health strategies, policy-makers can ensure that technological advancements do not merely supplement but actively transform accessibility and care delivery.^5,27^

###  Investing in Preventive Campaigns and Education 

 Given the public perception of oral health as a cosmetic rather than medical necessity,^28^ public health campaigns must focus on both individual behaviors and systemic awareness to shift these societal attitudes. Schools play a pivotal role in establishing lifelong oral health habits, and targeted campaigns can address caregivers and policy-makers, emphasizing the importance of oral health in systemic health and quality of life for vulnerable populations.

 Regular preventive campaigns for the elderly and disabled should highlight the systemic implications of oral health neglect. A multisectoral approach, including media campaigns, policy briefs, and community workshops, should be employed to raise awareness at multiple levels of society. Collaboration with national health agencies can enhance the sustainability and reach of these campaigns.^5,19^

###  Strengthening Research and Data Collection 

 Comprehensive data on oral health disparities and their economic impact is essential for evidence-based policy-making. Governments and health organizations should invest in tools for real-time data collection and analysis. Some evidences have provided valuable insights into the national and sub-national burden of oral diseases in Iran, highlighting areas where policy adjustments are needed.^1,10^

###  Empowering Volunteer Dental Professionals

 To expand the reach of oral health services, particularly in underserved and remote areas, it is vital to empower volunteer dentists and dental hygienists through structured training, standardized protocols, and supportive legal frameworks.^9^

 Although the overall number of dentists in Iran has increased, their distribution is uneven, and rural areas face shortages, making volunteer participation more challenging.^9^ To strengthen feasibility, incentives such as continuing education credits, official recognition, and logistical support can motivate professionals to contribute. In locations where no volunteers are available, mobile dental units and portable dental services should be deployed, while basic oral health promotion tasks can be integrated into community health worker programs.^24^

 These professionals can then act as critical agents in preventive education, early detection, and community-based care delivery. Integrating volunteer services into the national oral health infrastructure, supported by appropriate incentives, can significantly enhance coverage and sustainability of outreach efforts, especially for high-risk groups like the elderly and people with disabilities.^24^

## Conclusion

 Disparities in oral health persist, with elderly individuals and persons with disabilities continuing to experience systemic barriers in access to care. Improving oral health for special healthcare needed groups enhances quality of life and reduces the economic burden of untreated diseases. Coordinated national action is essential to close these gaps and implement effective policies.

###  Solutions Proposed

1. Integrate oral health into PHC systems.
2. Use digital tools to improve access and address workforce shortages.
3. Enhance oral health literacy through appropriate education.
4. Stakeholders collaboration is needed for sustainable, equitable solutions.

For clarity, these recommendations are summarized and ranked in Table.

**Table:** Summary of Policy Recommendations Ranked by Urgency and Feasibility

**Policy Recommendation**	**Urgency**	**Feasibility**	**Priority Level**
Integrate oral health into PHC	High	High	1
Use digital health tools (teledentistry, mobile apps)	High	Medium	2
Enhance oral health literacy and caregiver education	Medium	High	3
Strengthen research and data collection	Medium	Medium	4
Empower volunteer dental professionals	Medium	Medium	5

Abbreviation: PHC, primary healthcare.

## Takeaway Message

 To reduce oral health inequities, policy-makers should practically integrate oral care into PHC, leverage technology, and foster stakeholder collaboration. **The time to act is *NOW***!

## Acknowledgements

 We extend our sincere gratitude to all the organizations and individuals who supported this research. Our special thanks go to our colleagues, who have worked alongside us for years in serving patients requiring specialized care.

## Disclosure of artificial intelligence (AI) use

 Not applicable.

## Ethical issues

 The Project is approved by Research Ethics Committees of National Agency for Strategic Research in Medical Education (Approval ID: IR.NASRME.REC.1400.205).

## Conflicts of interest

 The authors have collaborated on various research projects for years. Both are interested in taking action to improve the level of health in Iran and both are concerned about the issue of oral health. This commonality and a sense of duty and commitment to the oral health of Iranians have led to the formation of this project and some related activities, including the launch of the Golkhand website (https://shcn.kmu.ac.ir).
